# A Novel Method of Extraction of Blend Component Structure from SANS Measurements of Homopolymer Bimodal Blends

**DOI:** 10.1002/macp.201300787

**Published:** 2014-04-03

**Authors:** Olga Smerdova, Richard S Graham, Urs Gasser, Lian R Hutchings, Davide S A De Focatiis

**Affiliations:** Division of Materials, Mechanics and Structures, University of NottinghamNottingham, NG7 2RD, UK; School of Mathematical Sciences, University of NottinghamNottingham, NG7 2RD, UK; Laboratory for Neutron Scattering, Paul Scherrer InstitutVilligen PSI, CH-5232, Switzerland; Department of Chemistry, University of DurhamDurham, DH1 3LE, UK

**Keywords:** bimodal blends, polystyrene, small-angle neutron scattering (SANS)

## Abstract

A new method is presented for the extraction of single-chain form factors and interchain interference functions from a range of small-angle neutron scattering (SANS) experiments on bimodal homopolymer blends. The method requires a minimum of three blends, made up of hydrogenated and deuterated components with matched degree of polymerization at two different chain lengths, but with carefully varying deuteration levels. The method is validated through an experimental study on polystyrene homopolymer bimodal blends with 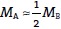. By fitting Debye functions to the structure factors, it is shown that there is good agreement between the molar mass of the components obtained from SANS and from chromatography. The extraction method also enables, for the first time, interchain scattering functions to be produced for scattering between chains of different lengths.

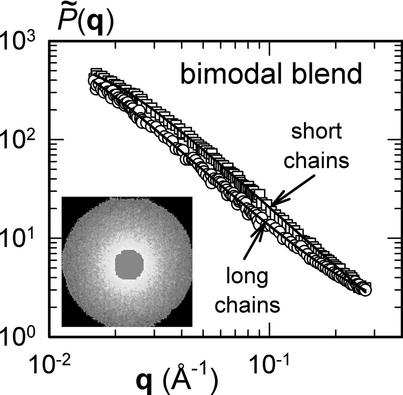

## 1. Introduction

Small-angle neutron scattering (SANS) has been proven as a powerful tool to study chain structure and dynamics of monodisperse polymers,^1^–^5^ blends,^6^–^9^ and polymer nanocomposites.^10^–^13^ SANS studies typically focus on the molecular structure of narrowly distributed molecular weights, of a single component within a blend of different polymers, or of crosslinked networks. In order to enhance scattering contrast, deuterated components with either matched degree of polymerization, or with intentionally differing molar mass,^14^ are introduced in a blend.

In spite of the substantial scientific interest in neutron scattering of polymers, interactions between polymer chains of different lengths within a homopolymer blend have not been studied previously. A method for obtaining quantitative information on these interactions would enable significant progress to be made toward successful modeling and prediction of all kind of properties of polydisperse polymers. In particular, it would facilitate a quantitative study on how established relaxation mechanisms such as Rouse retraction and reptation are influenced by chain length blending in oriented polymers.^5^

In principle, any polydisperse material can be represented as a discrete blend of a number of monodisperse components, each interacting with each other. From this point of view, understanding of the interaction between two molecular chain lengths in a bimodal blend during polymer flow is a crucial step towards elucidating the many interactions taking place in polydisperse polymers. Modeling frameworks for predicting solid-state properties of monodisperse melt-oriented polymers are prime candidates to benefit from this understanding, allowing developments to take place for the modeling of not only bimodal blends but eventually of commercial polydisperse resins.^15^,^16^

The aim of this publication is to develop and demonstrate a novel technique for the SANS study of bimodal blends that will allow quantitative structural information of the individual components to be obtained. A subsidiary benefit is that the technique also provides quantitative information on the interactions between different chain lengths within the blend. Two monodisperse polystyrenes of sufficiently but not substantially different molar mass 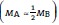 are selectively deuterated and blended together in different proportions but with the same chain length scale ratio to demonstrate the technique, in preparation for a wider study. The polymers used in this study and the experimental aspects of the SANS measurements are outlined first. The technique for the extraction of single-chain structure factors from bimodal blends is then developed from scattering theory, and applied to the measurements in order to obtain the single-chain form factors of both blend components, as well as the interchain scattering functions.

## 2. Experimental Section

### 2.1. Polymer Synthesis and Blending

Four samples, two of hydrogenated polystyrene and two of fully deuterated polystyrene, were synthesized by living anionic polymerization with benzene as the solvent and *sec*-butyllithium as the initiator using standard high-vacuum techniques.^17^ Molar mass was analyzed by size exclusion chromatography (SEC) on a Viscotek TDA 302 with refractive index, viscosity, and right-angle light-scattering detectors. The results of this analysis are presented in Table1. Hereafter, “*h*” and “d” correspond to hydrogenated and deuterated polystyrene components, respectively. A polydispersity index (PDI) less than 1.05 and a closely matched degree of polymerization *z* between deuterated and hydrogenated components were a criterion of bimodal blend quality.

**Table 1:** Molecular details of the monodisperse polystyrenes used for bimodal blend synthesis

Sample	Polymer	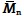 [kDa]	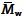 [kDa]	PDIa) [-]	*z*b) [-]
hA	hPS	91.6	93.8	1.025	881 ± 136
hB	hPS	214.4	223.4	1.040	2062 ± 422
dA	dPS	99.3	102.2	1.030	887 ± 152
dB	dPS	225.2	235.2	1.045	2011 ± 424

a) PDI is the polydispersity index, equal to 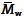/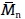;

b) *z* is the degree of polymerization, equal to 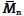/*M*_0_ where *M*_0_ is the monomer molar mass (104 Da for C_8_H_8_ hPS and 112 Da for C_8_D_8_ dPS), ± refers to the standard deviation.

Six bimodal blends with different composition of deuterated and hydrogenated polystyrenes, but identical fractions of short A and long B chains at a 4:1 mass ratio, were prepared from the monodisperse materials presented in Table1 by chemical blending in a solution followed by careful drying in a vacuum oven. The compositions of the six blends in term of the monodisperse components are summarized in Table2. The mass fractions of the deuterated polymer in the low- and high-molecular weight components are denoted as *x*_A_ and *x*_B_, respectively.

**Table 2:** Composition and deuterated fraction levels of the individual chain lengths for bimodal blends BL1–6

Blend	Composition				Deuterated fraction	
	dA [%]	hA [%]	dB [%]	hB [%]	*x*_A_ [-]	*x*_B_ [-]
BL1	76	4	16	4	0.95	0.80
BL2	76	4	8	12	0.95	0.40
BL3	68	12	16	4	0.85	0.80
BL4	76	4	12	8	0.95	0.60
BL5	72	8	12	8	0.90	0.80
BL6	72	8	16	4	0.90	0.60

### 2.2. Specimen Manufacture

All polystyrene specimens are manufactured by compression molding using a near net shape technique developed in our Nottingham laboratory.^16^ A mold with 11 identical rectangular cells of 100 mm × 6 mm × 1 mm is used to manufacture the isotropic bars of bimodal blends and monodisperse hydrogenated polymers. The mold, filled with the correct amount of polymer, was enclosed between two disposable aluminum sheets lightly coated with a PTFE release spray, and placed into a heated press at 170 °C.^18^ After a suitable holding time to ensure full relaxation and cooling at a rate of ca. 12 °C min^−1^ through to below the glass transition, the specimens were carefully removed from the mold, and trimmed to shape by cutting off any flash. Specimens were visually inspected for the absence of trapped air bubbles, and only bubble-free specimens were used in the study. Specimens molded in this way can be considered isotropic.

### 2.3. SANS Measurements

**Figure 1:**
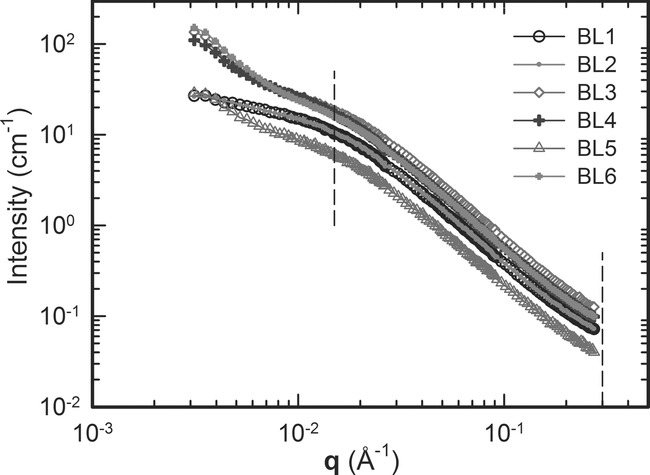
Measured scattering intensities obtained on specimens BL1–6. Dashed lines indicate the q range used in the least squares method. The solid lines are a guide to the eye.

The SANS study is based on experiments performed at the SANS-II station at the Swiss spallation neutron source SINQ, at the Paul Scherrer Institute, Villigen, Switzerland. The molded polystyrene bars were cut in two pieces and held together with adhesive tape in order to increase the scattering volume. The assemblies were attached individually to especially produced rectangular cadmium windows with an aperture width of 3 mm. Rectangular apertures were used in order to maintain consistency with a follow-up study of stretched bars, whose width was limited. Each assembly was fixed to an automatic sample changer. Using a neutron beam 8 mm in diameter produced an aperture of 3 mm × ca. 8 mm, and the total scattering volume was ca. 48 mm^3^. Differences in the scattering arising from the anisotropy of the instrument resolution are of the order of 1%. All experiments were performed at three separate sample-to-detector distances of 1.2, 4, and 6.15 m and neutron beam wavelengths of 0.475 and 1.05 nm in order to explore the scattering vector range from 0.005 to 0.3 Å^−1^. For all SANS measurements, data reduction and radial averaging was carried out with BerSANS software.^19^ Scattering curves for measurements at different scattering vector **q** ranges were superposed with ease. Each experimentally measured neutron-scattering intensity *I*(**q**) was normalized by the incident neutron flux, the solid angle of acceptance of the detector, the detector efficiency, and the scattering volume. The measured data were divided by the transmission of each sample. After this normalization, the subtraction of electronic noise determined in a measurement with cadmium blocking the incoming neutron beam, and subtraction of the background due to the sample holder, one obtains the scattering intensities shown in Figure 1, which can be interpreted and modeled theoretically. The reduced SANS data contain the coherent contribution of interest and an incoherent, flat background.

## 3. Results and Discussion

### 3.1. Scattering Theory for Bimodal Blends

A scattering model for bimodal blends is derived in order to interpret the experimental results. The purpose of developing this model is to enable the extraction of single-chain form factors from scattering intensities of bimodal blends. The proposed model is based on the theory detailed by Higgins and Benoît.^20^ A classical approach for interpreting neutron-scattering data from monodisperse polymers is to represent scattering intensity as composed of a single-chain form factor *P*(**q**) and an interchain scattering interference term *Q*(**q**). In monodisperse polymers, the application of an incompressibility condition allows the latter term to be removed, and the structure of polymer chains at a range of scattering wavelengths can be easily extracted. This approach was employed previously for blends of two (h,d) and three (h,h,d) monodisperse components, and for diblock copolymers.^6^,^21^ Bimodal blends investigated in this study contain four types of chains: short and long hydrogenated (subscripts hA and hB) and short and long deuterated (subscripts dA and dB). Thus the approach needs to be revised and extended for analysis of a four-component system.

The differential cross section for an *N*_s_-component polymer system in a reference polymer (designated by subscript 0) is given by:^20^

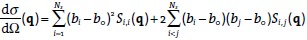
(1)where *b* is the scattering length and *S*(**q**) is the structure factor at the scattering vector **q**. In each of the four-component blends, the dominant component (by mass) is that of the short deuterated chains, dA. In the analysis that follows, the short deuterated chains are therefore considered as the reference polymer (and hence *b*_0_ = *b*_d_),^20^ although the analysis is independent of this choice. In Equation 1 the number of species *N*_s_ = 4. With *b*_0_ = *b*_d_, all terms where *b_i_* = *b*_d_ or *b_j_* = *b*_d_ vanish, and the following expression is obtained for the coherent differential cross section per unit volume:

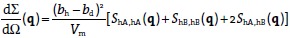
(2)

The monomer volume, which is a material constant, is *V*_m_. For each type of interaction, *S*(**q**) functions can be decomposed into two functions. For example, for the interaction between short–short hydrogenated molecules, the form factor for a single macromolecule *P*_hA,hA_(**q**) is given by:

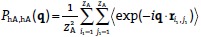
(3)where *z*_A_ is the degree of polymerization of the short mole­cules and **r** is a vector characterizing the position of the scattering point. The interchain interference between two points located on two different short chains *Q*_hA,hA_(**q**) is:

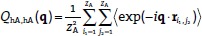
(4)

Both of these functions are normalized to unity at **q** = 0. There is a physical difference between them: while *P*(**q**) is intensive and dimensionless, *Q*(**q**) is extensive and inversely proportional to the number of molecules *N*.

The scattering function can be written as:^8^

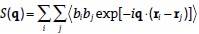
(5)where the sums run over all molecules in the sample. Each sum is split up into two sums over the *N* macromolecules and over the *z* monomers in each macromolecule:

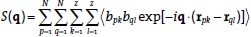
(6)

There are *N* contributions with *p = q* and *N*(*N −* 1) ≈ *N*^2^ with *p* ≠ *q*. The structure factor *S*_hA,hA_(**q**) can therefore be written in terms of inter- and intrachain scattering functions as:

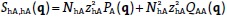
(7)

Using the same approach for other mixtures of chains, and making the necessary substitutions where necessary, *S*_hB,hB_(**q**) and *S*_hA,hB_(**q**) can be derived. The degree of polymerization of hydrogenated and deuterated chains of corresponding length is here close enough to be approximated as equal, setting *z*_hA_ ≈ *z*_A,_*z*_dA_ ≈ *z*_A_ and *z*_hB_ ≈ *z*_B,_*z*_dB_ ≈ *z*_B_. Substituting in appropriate expressions for the numbers of molecules *N*_dA_ = *x*_A_*N*_A_, *N*_hA_ = (1 − *x*_A_)*N*_A_, *N*_dB_ = *x*_B_*N*_B_ and *N*_hB_ = (1 −*x*_B_)*N*_B_, the general scattering intensity for a bimodal blend of partially deuterated polymers is obtained from Equation 2 and Equation 7 as follows:

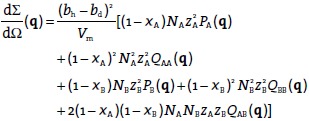
(8)

The Babinet principle, which states that the scattering of an object is equal to the scattering of the ‘negative’ of this object, was subsequently applied to the system. Considering a point *i* on molecule 1, a deuterated chain A, the scattering amplitude from all molecules except 1,*A*(**q**), is given by

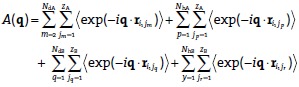
(9)

The scattering amplitude from molecule 1 is:

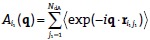
(10)

According to the Babinet principle, these two quantities must be equal in magnitude and opposite in sign. Summing Equation 9 and Equation 10 over all values of *i*_1_ and multiplying through by *N*_dA_ gives:

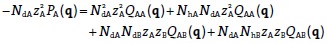
(11)

Substituting in expressions for the numbers of molecules as before, an additional equation relating form factors and inter-chain scattering function is obtained:

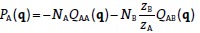
(12)

Using the same approach for a chain of type B, a second equation can be derived:

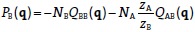
(13)

It is worth noting that these equations can also be derived from the incompressibility condition.

Substituting Equation 12 and 13 into Equation 8 eliminates from the scattering intensity two unknown functions *Q*_AA_(**q**) and *Q*_BB_(**q**). Therefore, the reduced scattering equation for the partially deuterated bimodal four-component blend is obtained as follows:

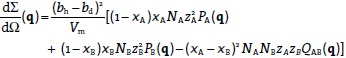
(14)

By this method, it is shown that the scattering intensity of the four-component system with long deuterated and hydrogenated chains with matched degrees of polymerization and short deuterated and hydrogenated chains with matched degrees of polymerization depends on only three independent functions: the form factor for chain A, the form factor for chain B, and the interchain interference function of A and B. Two further interchain interference functions between two short chains and between two long chains are not independent and are defined through Equation 12 and Equation 13, respectively.

In order to render the coefficients in Equation 14 dimensionless, the two form factors and three interchain interference factors are normalized by 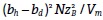 and 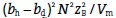 respectively, and the notations 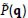 and 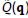 are used to denote the dimensionless terms. Here, *N* is the total number of molecules. Because the interchain interference functions *Q*(**q**) are extensive, they are normalized by *N*^2^ in order to make them independent of the system size. For the sake of simplicity, the following notation is also introduced: 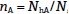, 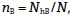, and 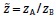. Therefore, Equation 12, 13, and 14 can be written as:

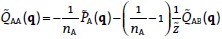
(15)

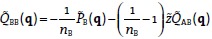
(16)

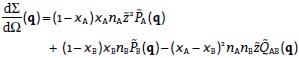
(17)

These reduced and normalized forms are used for the treatment of the scattering results presented in this communication. According to Equation 17, only three blends with varying levels of deuteration are, in principle, necessary to extract three independent structure functions and to characterize the molecular conformation.

An alternative approach is to employ standard random phase approximation (RPA) for the scattering cross section of an incompressible two-component blend:^22^

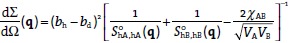
(18)where 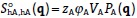 and 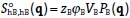 are non-interacting structure factors, and *χ*_AB_ is the Flory–Huggins interaction parameter between monomers A and B; *P*(**q**) are Debye functions. Several methods based on this approach were developed to calculate scattering of multicomponent incompressible and compressible polymer systems in matrix form.^23^,^24^ Other methods such as Ornstein–Zernike formalism and techniques based on the Edwards Hamiltonian were also applied for multicomponent polymer systems and are summarized by Vilgis et al.^25^ All these methods result in a matrix equation of the form 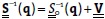, where 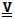 is an interaction matrix, which differs according to each approach. In contrast with these approaches, the technique presented in the present work allows interaction parameters 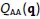, 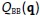

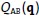
to be obtained as a function of the scattering vector **q**. The advantage is that it enables investigation of the polymer–polymer interactions across a range of length scales.

### 3.2. Application of the Scattering Theory

In order to make the best use of the available experimental data, the least squares method is applied to solve a linear system of equations corresponding to the experimental measurements on blends with matching degree of orientation. In each of the equations, the term 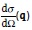 is the experimentally measured, normalized and radially averaged scattering intensity of every blend. The functions 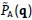, 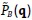 and 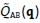 are treated as the unknown parameters of the model. The least squares solution is obtained by minimizing the difference between measured and calculated intensities from the obtained parameters at each measured **q** value. The parameter 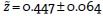 is used for all blends, while the parameters 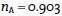 and 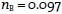 are calculated from 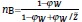 and 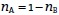, where *ϕ*_w_ is the weight fraction of long chains in a blend, equal to 0.2 for all the blends.

### 3.3. Internal Consistency and Error

The measured intensity curves were verified visually before applying the least squares method. After examination of the scattering curves, a **q** range of 0.015 Å^−1^ < **q** < 0.3 Å^−1^ was employed for all the calculations that follow. This was done because the scattering signal of some of the specimens at *q* < 0.015 Å^−1^ diverged from the shape normally expected for polymer systems. This divergence could have originated from undetected contamination of objects of a size >10 nm accumulated from the molding process, such as mold release, metal particles abraded from the mold tooling, or very small trapped air pockets. Although an individual correction could be applied to the scattering intensity of each blend, given the fact that specimens were synthesized, blended, dried, and subsequently molded using identical processes and that a single such correction could not be applied in the same way across all blends, it was deemed less arbitrary to impose a cut-off sufficiently far from this divergence in the analysis that follows. It must be recognized, however, that the presence of this contamination may have a small influence even at *q* > 0.015 Å^−1^.

In two of the six scattering measurements, the intensities were felt to be unreliable with respect to other measurements. They were measured on BL2 and BL5. Another possible reason for this discrepancy, besides potential contamination, is imperfect alignment of the specimen holder, and/or movement of the holder during the measurement, leading to a variation of scattering volume. Since sufficient other measurements were available to perform the least squares calculation with redundancy, measurements BL2 and BL5 were removed from the analysis.

After applying the least squares method, the internal consistency of the system is verified by the inverse calculation of the intensity functions from the form factors and the interchain interference functions for all blends, using Equation 17. The normalized root mean square error arising from the application of this procedure to the differential cross section of all blends was calculated for each value of **q** from the form factors and interchain interference functions by the proposed method, with summation over *N*_B_ blends at each value of **q**. The average magnitude of this error is 10.1%. The error is statistically independent of the scattering vector value. It should be pointed out that although this error may at first sight appear large, most SANS studies do not have the redundancy required to calculate the errors at all. The relatively moderate and known magnitude of the error level demonstrates that the proposed method is a reliable tool to extract the single-chain factors and the interchain interference functions from partially deuterated bimodal blends.

### 3.4. Structure Form Factors

**Figure 2:**
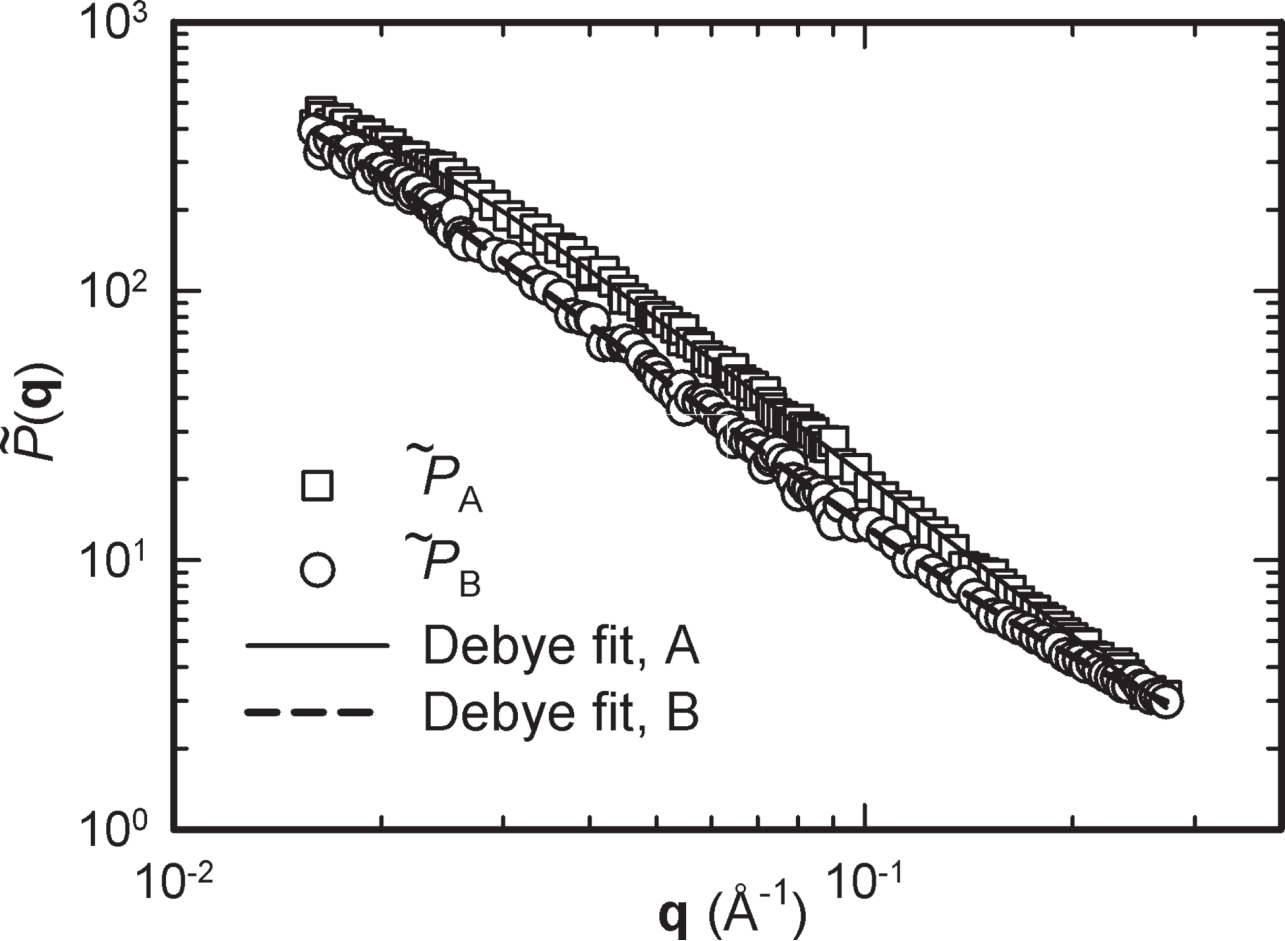
Normalized structure form factors 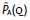, 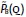 of low- and high-molecular-weight components within the same blend. Data extracted from SANS using the least squares technique is shown as symbols. Debye functions are fitted to these measurements and shown as lines.

For ideal Gaussian chains of monodisperse polymers, the structure form factor is usually modeled using functions of the form:^4^

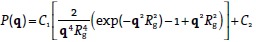
(19)where *C*_1_ and *C*_2_ are constants, and the term in square brackets is a Debye function^26^ in which *R*_g_ is the radius of gyration of a polymer chain. The approach applied here is to treat the three parameters *C*_1_, *C*_2_, and *R*_g_ as fitting coefficients to obtain *P*(**q**). The resulting functions, as well as the experimentally measured form factors 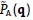, 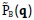 extracted by the method described above for isotropic materials, are presented in Figure 2, and values of the fitting coefficients and errors calculated by the bootstrap method are reported in Table3.

**Table 3:** Fitting coefficients to the structure form factors, mean weight-averaged molar mass of the four blend components obtained from SEC, and molar mass obtained from extraction of the radius of gyration from SANS experiments, for blend components A and B

Blend component	Fitting coefficientsa)			Molar mass from SEC	Molar mass from SANS
	*C*_1_ [-]	*C*_2_ [-]	*R*_g_ [Å]	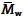 [kDa]	(*R*_g_/C)^2^ [kDa]
A	783.3 ± 6.2	0.9 ± 0.5	87.2 ± 0.6	101.4	96.9–108.2
B	1239.8 ± 16.9	1.9 ± 0.6	142.8 ± 1.3	232.2	260.1–290.4

a) Errors on the fitting coefficients were obtained via the bootstrap method. First, both form factors were simply fitted with three unknown parameters in order to determine *C*_1_, *C*_2,_ and *R*_g_. The error on *R*_g_ was found keeping *C*_1_ and *C*_2_ values constant and taking 1000 random datasets from the first 20 scattering data points at low **q**. Then, the value of *R*_g_ was kept constant while *C*_1_ and *C*_2_ were fitted to 1000 datasets from the whole scattering range.

The radii of gyration for short and long chains, equal to 87.2 and 142.8 Å respectively, allow a calculation of molar mass based on *R*_g_ from the relationship:^27^

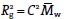
(20) using a value of *C* in the range of 0.265–0.280 for atactic polystyrene in glass state.^28^ A comparison with the weight-average molar mass of the components is shown in Table3. There is an excellent agreement between mean values of the weight-averaged molar mass measured by SEC and obtained from extraction of the structure form factors for component A, and relatively good agreement for component B. It must be noted that component B is present in much lower concentration in the bimodal blend, and that the radius of gyration of component B is relatively high compared with the **q** measurement range, introducing expected inaccuracies in the determination of *R*_g_. Nevertheless, the good agreement is further confirmation that the extraction technique successfully enables analysis of individual blend components within a blend, whether isotropic or oriented.

### 3.5. Interchain Scattering Functions

**Figure 3:**
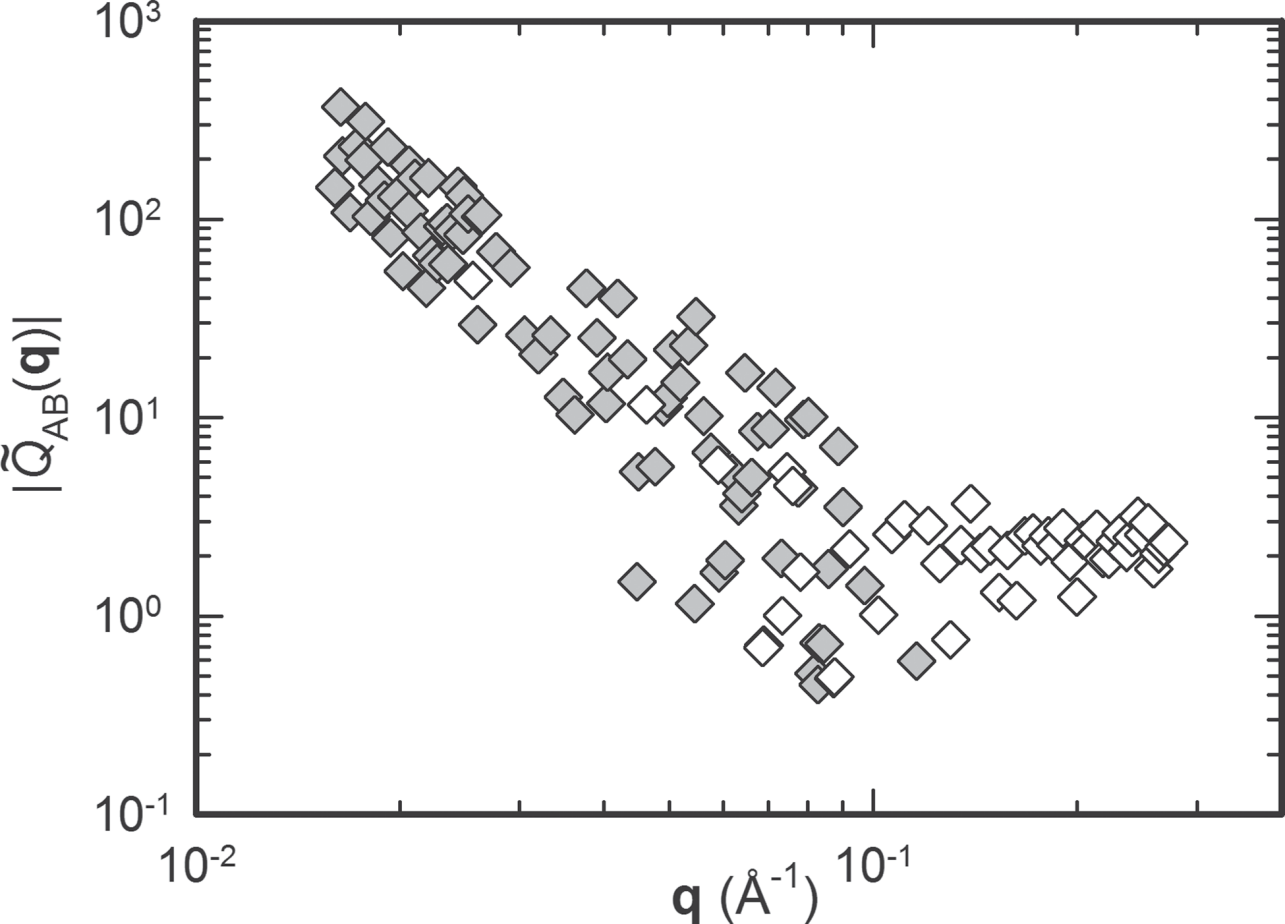
Absolute value of the normalized interchain scattering function 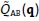 for scattering between short and long chains within the same blend. Gray shading indicates a negative sign.

**Figure 4:**
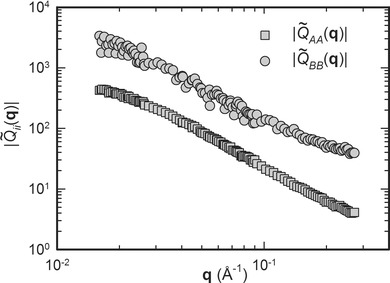
Absolute value of the normalized interchain scattering functions 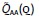 and 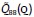 for scattering between chains of the same length within the same blend. Gray shading indicates a negative sign.

One of the unique features of this method is its ability to reveal the interchain scattering function between two chains of different molar mass, and hence different length, within the same blend. To the authors' knowledge, this has not been shown before in the context of a homopolymer blend. Figure 3 displays the magnitude of 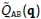, the normalized interchain scattering function for scattering between chains of different lengths, obtained from the least squares analysis. The function is broadly similar in shape to the structure factors, but changes in sign from negative to positive at **q** ≈ 0.1 Å^−1^ at small intensities. From Equation 15 and 16 it is also possible to obtain the normalized interchain scattering functions for scattering between different chains of the same length, 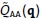 and 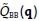 and the absolute values of these functions are shown in Figure 4. The values of these functions are negative throughout the **q** range. It is hoped that the availability of these functions will spur further development of theory and models in this area.

## 4. Conclusion

This work has proposed a novel method of extracting single-chain form factors from the scattering intensity functions of a homopolymer blend composed of two distinct chain lengths. The least squares method was demonstrated for extracting two single-chain form factors and three interchain interference functions. Although in principle just three blends with different deuteration levels are sufficient to complete this extraction, data from four blends were used here in order to obtain an estimate of the error in the procedure, found to be 10.1%. The method was validated through an experimental study carried out on selectively deuterated polystyrene blends. By fitting Debye functions to the single-chain form factors, it was possible to determine radii of gyration, and hence molar mass measurements of the two components within the blend. These compared well to measurements obtained prior to blending using chromatography. The method also allows, for the first time, to determine the interchain scattering function between two chains of different molar mass, and hence different length, within the same blend.

## References

[b1] Casas F, Alba-Simonesco C, Montes H, Lequeux F (2008). Macromolecules.

[b2] Muller R, Picot C, Zang YH, Froelich D (1990). Macromolecules.

[b3] Blanchard A, Graham RS, Heinrich M, Pyckhout-Hintzen W, Richter D, Likhtman AE, McLeish TCB, Read DJ, Straube E, Kohlbrecher J (2005). Phys. Rev. Lett.

[b4] Graham RS, Bent J, Hutchings LR, Richards RW, Groves DJ, Embery J, Nicholson TM, McLeish TCB, Likhtman AE, Harlen OG, Read DJ, Gough T, Spares R, Coates PD, Grillo I (2006). Macromolecules.

[b5] Bent J, Hutchings LR, Richards RW, Gough T, Spares R, Coates PD, Grillo I, Harlen OG, Read DJ, Graham RS, Likhtman AE, Groves DJ, Nicholson TM, McLeish TCB (2003). Science.

[b6] Benmouna M, Briber R, Hammouda B (1997). Macromol. Theory Simul.

[b7] Hammouda B, Briber RM, Bauer BJ (1992). Polymer.

[b8] Warner M, Higgins JS, Carter AJ (1983). Macromolecules.

[b9] Graham RS, Bent J, Clarke N, Hutchings LR, Richards RW, Gough T, Hoyle DM, Harlen OG, Grillo I, Auhl D, McLeish TCB (2009). Soft Matter.

[b10] Genix AC, Tatou M, Imaz A, Forcada J, Schweins R, Grillo I, Oberdisse J (2012). Macromolecules.

[b11] Nishida T, Obayashi A, Haraguchi K, Shibayama M (2012). Polymer.

[b12] Nusser K, Schneider GJ, Pyckhout-Hintzen W, Richter D (2011). Macromolecules.

[b13] Chua YC, Chan A, Wong HC, Higgins JS, Cabral JT (2010). Macro­molecules.

[b14] Tassin JF, Baschwitz A, Moise JY, Monnerie L (1990). Macromolecules.

[b15] De Focatiis DSA, Embery J, Buckley CP (2010). J. Polym. Sci., Part B: Polym. Phys.

[b16] De Focatiis DSA, Buckley CP (2012). J. Polym. Sci., Part B: Polym. Phys.

[b17] Clarke N, Colley FR, Collins SA, Hutchings LR, Thompson RL (2006). Macromolecules.

[b18] De Focatiis DSA, Buckley CP (2008). Polym. Test.

[b19] Keiderling U (2002). Appl. Phys. A: Mater. Sci. Process.

[b20] Higgins JS, Benoît H (1994). Polymers and Neutron Scattering.

[b21] Leibler L (1982). Macromolecules.

[b22] de Gennes PG (1979). Scaling Concepts in Polymer Physics.

[b23] Ziya Akcasu A, Tombakoglu M (1990). Macromolecules.

[b24] Kim JK, Kimishima K, Hashimoto T (1993). Macromolecules.

[b25] Vilgis TA, Benmouna M, Benoit H (1991). Macromolecules.

[b26] Debye P (1947). J. Phys. Colloid Chem.

[b27] Flory PJ (1949). J. Chem. Phys.

[b28] Wignall GD, Melnichenko YB (2005). Rep. Prog. Phys.

